# Self-assembly of nanostructures and nanomaterials

**DOI:** 10.3762/bjnano.6.144

**Published:** 2015-06-24

**Authors:** Isabelle Berbezier, Maurizio De Crescenzi

**Affiliations:** 1 CNRS-AMU, Im2np, Faculté des Sciences et Techniques, Avenue Escadrille Normandie Niemen, 13397 Marseille Cedex 20, France; 2 Department of Physics, University of Roma Tor Vergata, Via della Ricerca Scientifica, 1, 00133 Roma, Italy

**Keywords:** nanomaterials, nanostructures, self-assembly phenomena

Presently, most microelectronic devices are fabricated using top-down approaches. According to Moore’s law, with the predicted ultimate reduction in size over the next years, these processes will reach the limits of technological instrument resolution. In addition, a major bottleneck in top-down approaches is the prohibitive cost due to the large number of technological steps required to reduce the device size. Furthermore, a broad range of applications require ultrasmall, complex devices that cannot be produced using top-down methods. New processes building on the natural self-organization of matter should therefore be conceived and developed, along with adequate characterization methods in order to allow for their application in innovative devices. Such self-organization processes are already implemented in various materials such as biological materials, soft matter, metals and semiconductors. Self-assembly is a process that builds an ordered structure, brick-by-brick, starting from disordered building blocks, using simple key ingredients. Self-assembly is commonly controlled by certain intrinsic material parameters (e.g., composition, strain, thickness, phase transformation, structural changes) and results from the interaction between different factors (e.g., deposit/substrate, liquid/gas/solid phases, crystals). Besides these intrinsic parameters, a number of extrinsic factors, including thermal treatment, chemical and electrochemical reactions, mechanical stress, electric or magnetic fields, can strongly influence the self-assembled morphologies.

Self-assembly processes are generally low cost, large-scale techniques, which can be suitable for various industrial environments. Identified as one of the key topics in nanoscience with potential to shape future scientific research, self-assembly is the most promising path to breakthroughs in nanoelectronics, optoelectronics, spintronics, molecular nanotechnology, biology, materials science, software, robotics, manufacturing, transportation, energy harvesting, infrastructure and construction.

Research on self-assembled nanostructures encompasses fundamental issues in chemical synthesis, crystal growth and self-organization of 0D, 1D and 2D nanostructures, nanopatterning, lithographic techniques, nanocharacterization, scaling of materials properties down to molecular dimensions, quantum properties and applications of nanoscale assemblies to advanced devices. The main topics of interest involve 2D nanomaterials such as nanomembranes, graphene, silicene and ordered mesoporous oxides, 1D nanomaterials such as nanowires and nanotubes, and 0D nanomaterials such as quantum dots, nanocrystals and Q-bits.

Focusing on this dynamic new field, self-assembly “the science of things that put themselves together” explores the physics of nanostructures, new synthesis approaches, in addition to size-, shape- and composition-dependent properties. The major obstacles concern the reproducibility and control of the basic mechanisms in order to predict and produce patterns with tunable size, periodicity and position and the new physical properties resulting from low dimensionality.

The goal of this Thematic Series is to bring together studies in the broad field of research on self-assembled nanostructures, which may seem far away, but are expected to promote an extraordinary exchange of ideas appropriate to conceive multidisciplinary processes suitable for various materials with the aim to produce exceptional new nanomaterials with revolutionary properties.

This Thematic Series covers the physics of nanostructures at the nanoscale including:

- large-scale patterning obtained by spontaneous structuring as well as local probe nanopatterning for nanostructure size and position control;
- theoretical and experimental efforts dedicated to a better understanding of the formation, evolution, and organization of nanoscale systems;
- fundamental and new issues in nucleation, crystal growth, surface and interface atomistic mechanisms; and
- new optical, electrical, magnetic, and mechanical properties of self-assembled systems.

Isabelle Berbezier and Maurizio De Crescenzi

Marseille and Rome, June 2015

